# Patterns and determinants of plant biodiversity in non-commercial forests of eastern China

**DOI:** 10.1371/journal.pone.0188409

**Published:** 2017-11-21

**Authors:** Chuping Wu, Mark Vellend, Weigao Yuan, Bo Jiang, Jiajia Liu, Aihua Shen, Jinliang Liu, Jinru Zhu, Mingjian Yu

**Affiliations:** 1 College of Life Sciences, Zhejiang University, Hangzhou, Zhejiang, China; 2 Zhejiang Academy of Forestry, Hangzhou, Zhejiang, China; 3 Departement de biologie, Université de Sherbrooke, Sherbrooke, QC, Canada; Chinese Academy of Forestry, CHINA

## Abstract

Non-commercial forests represent important habitats for the maintenance of biodiversity and ecosystem function in China, yet no studies have explored the patterns and determinants of plant biodiversity in these human dominated landscapes. Here we test the influence of (1) forest type (pine, mixed, and broad-leaved), (2) disturbance history, and (3) environmental factors, on tree species richness and composition in 600 study plots in eastern China. In total, we found 143 species in 53 families of woody plants, with a number of species rare and endemic in the study region. Species richness in mixed forest and broad-leaved forest was higher than that in pine forest, and was higher in forests with less disturbance. Species composition was influenced by environment factors in different ways in different forest types, with important variables including elevation, soil depth and aspect. Surprisingly, we found little effect of forest age after disturbance on species composition. Most non-commercial forests in this region are dominated by species poor pine forests and mixed young forests. As such, our results highlight the importance of broad-leaved forests for regional plant biodiversity conservation. To increase the representation of broad-leaved non-commercial forests, specific management practices such as thinning of pine trees could be undertaken.

## 1. Introduction

Balancing economic development and biodiversity conservation is a major challenge for forest management, especially in developing countries [1, 2]. To address this issue, many countries have initiated forest management programs that distinguish different forest types, such as commercial forest and non-commercial forest [3, 4]. Non-commercial forest is protected from human intervention with a focus on the maintenance of forest ecosystem functions (e.g., carbon fixation, water filtration) and biodiversity, while commercial forest is the source of timber consumption. Non-commercial forests are under changing management regimes and might play an important role in biodiversity conservation in human dominated landscapes [5].

Non-commercial forest occupies a large area globally. For example, the area of non-commercial forest in China is roughly 85 million ha (about 41.6% China’s forest land) [6], consisting largely of secondary forests and plantations [2], such as Masson pine (*Pinus massoniana*) forests, which are widely distributed in China [7]. Many planted and natural forests are dominated by just one or a few tree species that are deemed to have relatively low conservation value compared to primary forests [8, 9]. In order to improve biodiversity and ecosystem functions of monocultures and young forests via acceleration of succession, management actions include thinning, planting of broad-leaved trees, and reducing competition around (i.e., singling) broad-leaved individuals [10, 11]. For example, Meng et al. [10] used thinning and enrichment planting to transform a degraded Masson pine plantation to a mixed forest with increased species diversity. However, no studies have compared biodiversity in different types of non-commercial forests in China, especially at broad scales.

Multiple factors can potentially affect biodiversity in non-commercial forests, which are products of natural environmental variation and past anthropogenic activities. Firstly, as found in most studies on primary forests, biodiversity patterns can be influenced by environmental factors [12–18], such as soil and topography. Secondly, dominant canopy trees (captured in forest type classifications) can be an important determinant of species diversity in the rest of the community [19], while also being related to environmental gradients. In eastern China, pine forest, mixed forest, and broad-leaved forest are the main types of non-commercial forest. Thirdly, disturbance history can have a strong influence on species richness, community structure and species composition [20, 21]. For example, as succession proceeds after disturbance, late successional tree species typically increase in abundance as early successional species decline, gradually converging on the species composition of mature forests [22, 23]. These shifts of biodiversity patterns in terms of plant functional traits, species composition and ecosystem function are often predicted by the age since abandonment [24]. Most non-commercial forests in China have regenerated since the 1960s, with variable disturbance histories, but how these disturbances influence biodiversity patterns are rarely studied.

Due to limited datasets combining information on all of these anthropogenic and environmental factors, we still lack a systematic understanding of biodiversity patterns and their determinants in non-commercial forests of China. In this paper, we present an analysis exploring the patterns and determinants of tree biodiversity in non-commercial forests in eastern China. We chose Zhejiang province as a case study as this region is mainly dominated by non-commercial forest, and local governments have made major investments in forest management for conserving biodiversity and ecosystem functioning. We aimed to test the influence of (1) forest type (pine, mixed, and broad-leaved), (2) forest age after disturbance and (3) environmental factors, on tree species diversity and composition among survey plots. The results can provide scientific guidance to the management of non-commercial forests in this region.

## 2. Material and methods

### 2.1 Study area

**Fig 1:**
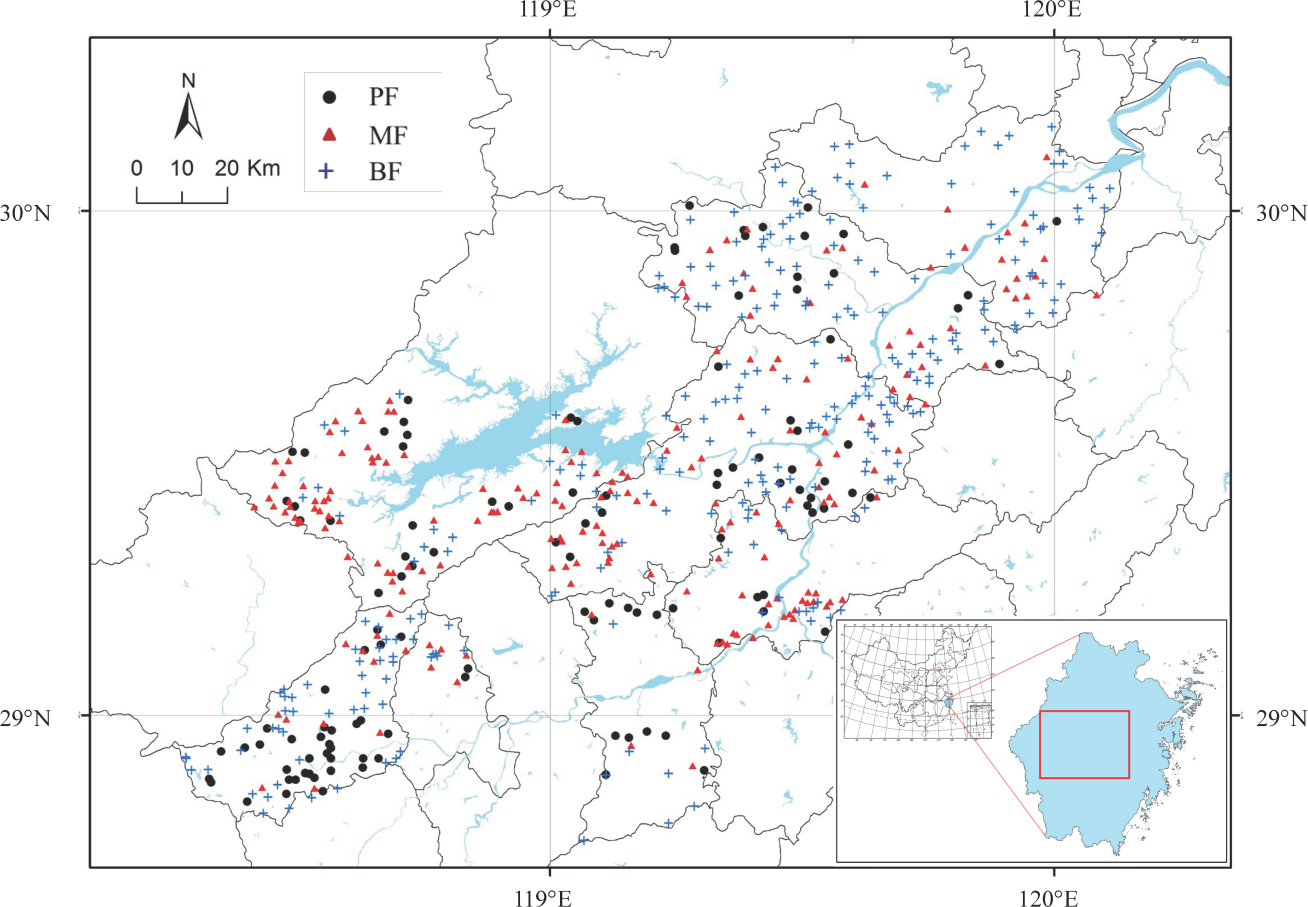
Geographic distribution of survey plots. PF: pine forest; MF: mixed forest; BF: broad-leaved forest. China map is from http://www.tianditu.gov.cn/.

Our study was conducted in the subtropical forest region of eastern China (Fig 1). The region experiences a subtropical monsoon climate with mean annual precipitation of 980–2000 mm, and mean annual temperature of 15–18°C. July mean temperature maximum ranges from 33–43°C, and February mean minimum temperature ranges from -2.2 to -17.4°C (data from www.zj.gov.cn). Zonal mature vegetation in this region is mainly subtropical evergreen broad-leaved forest, in which evergreen broad-leaved trees dominate in the canopy layer [25]. Non-commercial forests of Zhejiang covered roughly 2.68 million ha in 2014, accounting for 25.7% of the total land area (10.41 million ha) and 40.6% of the forested land area (6.60 million ha). According to a 2010 report [26], pine-dominated forest accounted for 35.5% of the forested area, mixed broad-leaved-conifer forest 13.7%, broad-leaved forest 28.5%, and other forest types, such as Chinese fir, bamboo forest, and shrub forest, 22.3%.

### 2.2 Study design

To monitor forest vegetation, the Forestry Department of Zhejiang province set up and surveyed 600 plots, each 20 × 20 m, in non- commercial forests from 2010 to 2015 (Fig 1). These plots were evenly distributed across the study region and reflected the main stand types characteristic of the local vegetation. All trees with diameter at breast height (DBH) ≥ 5 cm were identified and measured. Each plot was first classified as one of three types (pine forest, mixed forest, broad-leaved forest) using importance values (IV), calculated for a given species as IV = 100 × (relative density + relative frequency + relative basal area)/3. Relative frequency in every 20 × 20 m plot was calculated from 16 subplots (5 × 5 m). If the IV for pine was > 66.7%, the plot was classified as pine forest (n = 169 plots); if it was between 33.3% and 66.7%, the plot was classified as mixed forest (n = 170); if < 33.3% (i.e., the summed IV of broad-leaved species was > 66.7%), the plot was defined as broad-leaved forest (n = 261). Each plot was further classified according to forest age–stage 1 (pine forest: 30 plots, mixed forest: 32 plots, broad-leaved forest: 96 plots), stage 2 (46, 58, 125), and stage 3 (93, 80, 40)–based on the average age of the dominant trees in the plot (Table 1). Stage 1 stands are <20 years old, stage 2 stands 20–60 years old, and stage 3 stands >60 years old. Forest age after disturbance was estimated based on the records of cutting history in a given plot. In addition to tree composition data, for each plot we also estimated total canopy density, soil depth, humus depth, litter depth, elevation, slope, slope position, and aspect. Canopy density was estimated as the mean of visual estimates at 48 points per plot, with three points randomly set in each of the sixteen 5 × 5 m subplots. We also measured slope, aspect, elevation, longitude and latitude by compass and GPS.

**Table 1:** Total number of plots, plant families, genera and species of trees in different forest types.

Forest types	Number of plots				Families	Species
	Stage1	Stage2	Stage3	Total		
Pine forest	30	46	93	169	32	70
Mixed forest	32	58	80	170	48	116
Broad-leaved forest	96	125	40	261	50	128
Total	158	229	213	600	53	143

### 2.3 Data analyses

In order to explore variation in species composition in different forest types, we first report importance values of the tree species recalculated by combining all plots of a given forest type. The relative frequency in a given forest type was calculated for all 20 × 20 m plots of that forest type.

We used one-way ANOVAs to test for differences in species richness among forest types. A generalized linear model was conducted to predict species richness as a function of environmental variables (i.e. forest age, canopy density, soil depth, humus depth, litter depth, elevation, slope, slope position, aspect). Before GLM analysis, we implemented a test for overdispersion using the function of dispersiontest in the R package AER [27]. Because our data showed significant overdispersion, we used a negative binomial error distribution with a log link function for species richness (count data). This analysis was performed using the “glm.nb” function from the MASS package in R Version 3.2.4 (R Core Team, 2016). To determine which of these explanatory variables were most important in predicting species richness, we used the “dredge” function in the MUMIN package, which is used to identify significant predictors in multiple models, to test all possible combinations of the environmental variables; we then used Akaike’s information criterion (AICc), corrected for small sample sizes, to select the best model [28]. We calculated a commonly used index, i.e. the Bray-Curtis index, as a metric of compositional dissimilarity between all pairs of plots [29, 30], as the input for non-metric multidimensional scaling (NMDS) ordinations (with 999 iterations), one for each forest type. These analyses were performed using the function “metaMDS” in the vegan package of R. In order to focus on the dominant components of the vegetation, rare species (<10 stems) were removed prior to the NMDS analyses, because their occurrences in the dataset can depend on chance and potentially distort the ordination [31, 32]. The significance of environmental variables in predicting species composition were tested using the function “envfit” in the vegan package [33].

## 3. Results

### 3.1 Species richness in different forest types

Overall, across all plots we found 143 species in 53 plant families. Broad-leaved forests had the greatest total diversity, with 128 species and 50 families, followed by mixed forests and pine forests (Table 1).

**Fig 2:**
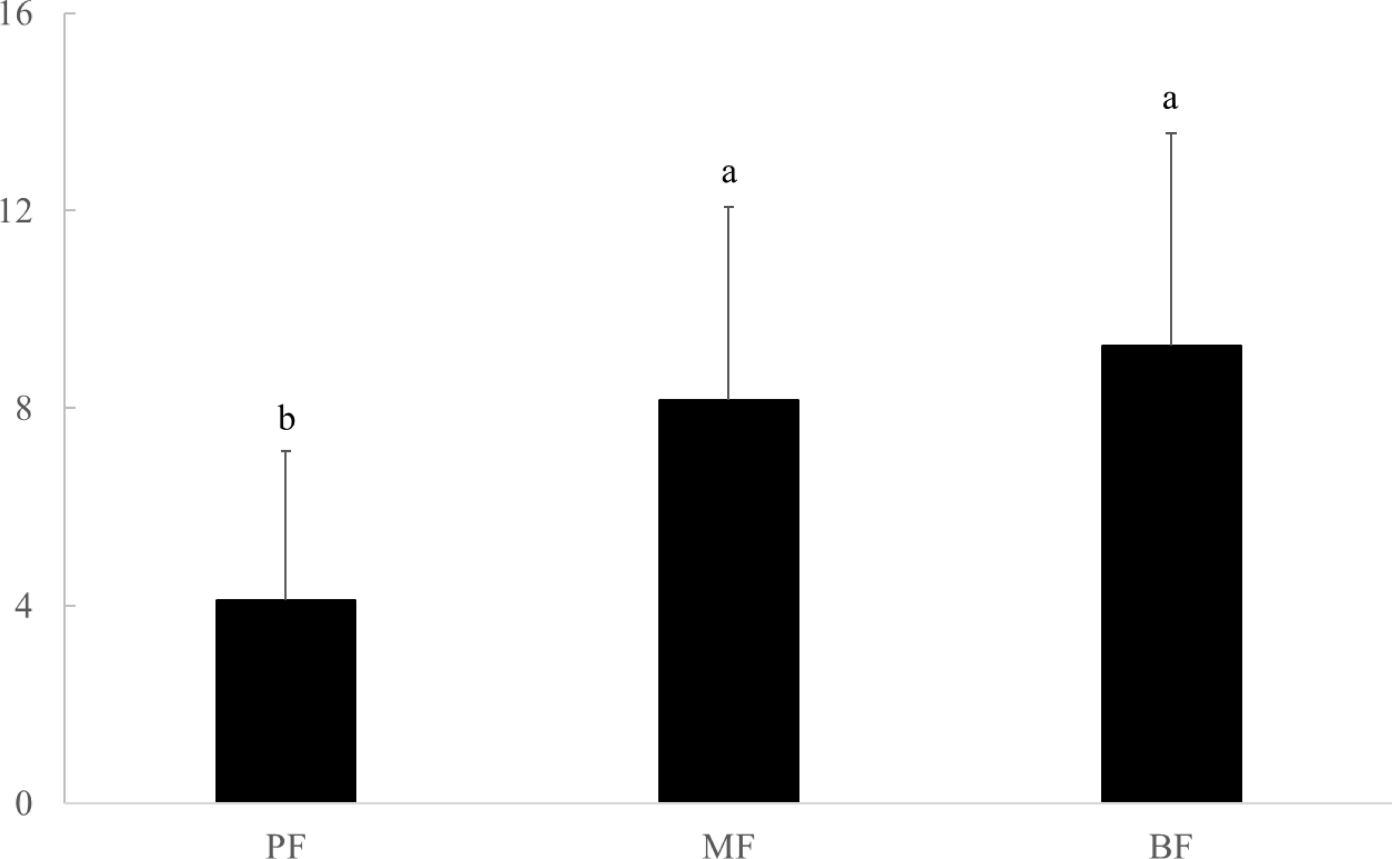
Species richness per plot in three forest types. PF: pine forest; MF: mixed forest; BF: broad-leaved forest.

Species richness per plot was greater in mixed forest and broad-leaved forest than in pine forest (Fig 2). Species richness was qualitatively greater in broad-leaved forest than in mixed forest, but the differences were not significant. The best predictors of species richness were elevation and forest age in mixed forest and broad-leaved forest, and canopy density in pine forest (Table 2).

**Table 2:** Results of a generalized linear model predicting species richness in each forest type as a function of environmental variables (i.e., forest age, canopy density, soil depth, humus depth, litter depth, elevation, slope, aspect, slope position).

Forest types	Predictors	Estimate	SE	z	*P* value
Pine forest	Canopy density	1.431	0.366	3.908	<0.001***
Mixed forest	Elevation	0.001	0.0001	3.280	0.010**
	Forest age	0.093	0.051	1.845	0.065
Broad-leaved forest	Elevation	0.001	0.0001	4.950	<0.001***
	Forest age	0.010	0.003	3.112	0.013*
	Humus depth	-0.038	0.022	-1.743	0.081

SE: standard error of estimates. z: Wald statistic for testing the hypothesis that the corresponding estimate is equal to zero (null hypothesis).

*P < 0.05.

** *P* < 0.01.

*** *P* < 0.001.

### 3.2 Species composition

**Fig 3:**
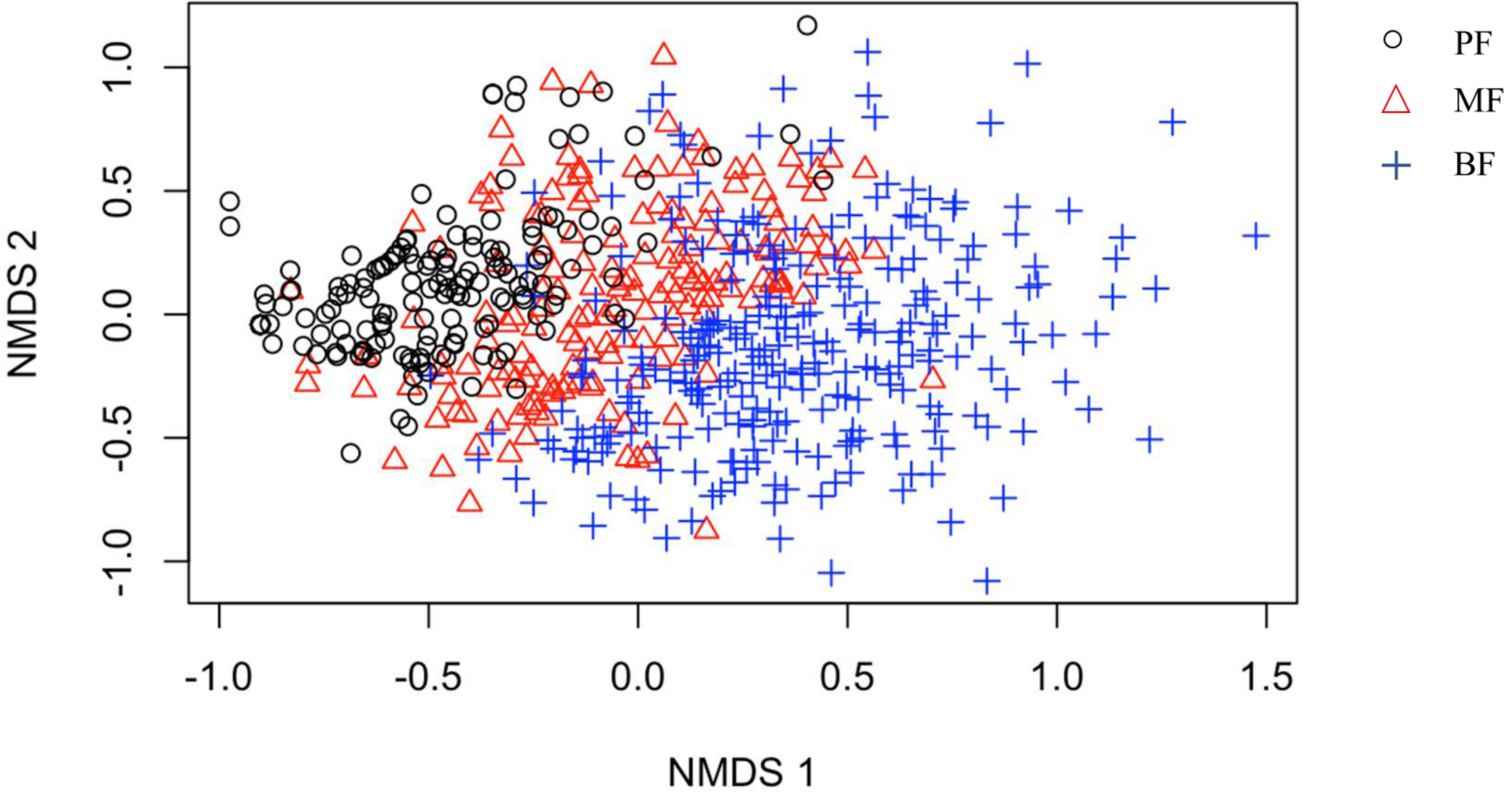
Two dimensional Non-Metric Multidimensional Scaling (NMDS) ordination diagram of all forest types together. PF: pine forest; MF: mixed forest; BF: broad-leaved forest.

The NMDS analysis revealed clear distinctions in tree species composition among the three forest types, as expected given that forest types are defined by their dominant trees (Fig 3). The average importance value (IV) of conifers such as *Pinus massoniana* was 70.1%, 40.8%, and 9.8% in pine forest, mixed forest and broad-leaved forest, respectively. The broad-leaved trees in pine forest were largely early successional species, including deciduous broad-leaved trees (e.g., *Quercus fabri*, *Liquidambar formosana*), and evergreen broad-leaved trees (e.g., *Schima superba*, which is early successional species and can grow in late stages). Deciduous broad-leaved trees and evergreen broad-leaved trees had similar dominance levels in mixed forest. Evergreen broad-leaved trees dominated in broad-leaved forest (Table 3).

**Table 3:** Importance values in each forest type of the 10 most common tree species.

	Species	Important value (%)		
		PF	MF	BF
Coniferous trees	*Pinus massoniana*	63.8	32.9	4.9
	*Cunninghamia lanceolata*	6.3	7.9	4.9
Deciduous broad-leaved trees	*Dalbergia hupeana*		2.0	
	*Liquidambar formosana*	2.1	2.6	4.0
	*Loropetalum chinense*	1.3	2.8	
	*Quercus acutissima*		2.1	2.8
	*Quercus fabri*	2.8	4.3	8.7
Evergreen broad-leaved trees	*Castanopsis eyrei*			3.4
	*Castanopsis sclerophylla*	1.5	3.0	5.0
	*Cinnamomum platyphyllum*	1.9		
	*Cyclobalanopsis glauca*		3.7	10.6
	*Lithocarpus glaber*			3.1
	*Schima superba*	1.4	5.1	8.9

Here importance values were computed by first combining all plots of a given forest type. PF: pine forest; MF: mixed forest; BF: broad-leaved forest.

### 3.3 Influence of environmental factors on species composition

**Fig 4:**
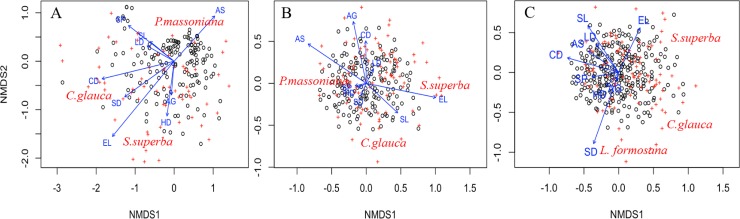
NMDS ordinations of species composition and environmental factors in three forest types. A: pine-dominated forest; B: mixed broad-leaved-conifer forest; C: broad-leaved forest. SL: slope; SP: slope position; AS: aspect; EL: elevation; SD: soil depth; HD: humus depth; LD: litter depth; CD: canopy density; AG: age.

We analyzed the influence of environmental factors on species composition in each forest type separately (Fig 4, Table 4). In broad-leaved forests, soil depth (*R*^2^ = 0.074, *P* = 0.001) was the best predictor of species composition followed by elevation (*R*^2^ = 0.061, *P* = 0.001), canopy density (*R*^2^ = 0.056, *P* = 0.002), slope (*R*^2^ = 0.050, *P* = 0.003), litter depth (*R*^2^ = 0.026, *P* = 0.025), and aspect (*R*^2^ = 0.024, *P* = 0.044). Species composition of mixed forests was significantly affected by elevation (*R*^2^ = 0.050, *P* = 0.023), and aspect (*R*^2^ = 0.044, *P* = 0.031). Species composition in pine forests was explained by elevation (*R*^2^ = 0.064, *P* = 0.002) and canopy density (*R*^2^ = 0.046, *P* = 0.028).

**Table 4:** Significant correlations of environmental variables with NMDS axes.

Forest type	Environmental variable	r^2^	*P* value
Pine forest	Elevation	0.0644	0.005**
	Canopy density	0.0464	0.018*
Mixed forest	Aspect	0.0444	0.033*
	Elevation	0.0500	0.019*
Broad-leaved forest	Slope	0.0470	0.001***
	Elevation	0.0354	0.009**
	Soil depth	0.0804	0.001***
	Canopy density	0.0500	0.005**

NMDS analyses were based on the matrix of species dissimilarities (Bray-Curtis index).

* *P* < 0.05.

** *P* < 0.01.

*** *P* < 0.001.

## 4. Discussion

### 4.1 Biodiversity in non-commercial forests

To improve biodiversity conservation in China’s forests, non-commercial forests have been protected from anthropogenic disturbances and currently occupy 41.6% of China’s forested land [6]. Our study, to our knowledge, is the first to investigate biodiversity patterns in non-commercial forests at the regional level with a large dataset. In total, we found 143 species across 600 plots. These forests harbor some rare species, e.g. *Emmenopterys henryi* and *Cercidiphyllum japonicum* (both are Grade II of National Key Protected Wild Plants) and some commercially valuable species, e.g. *Phoebe sheareri* and *Zelkova serrata*. In addition, these non-commercial forests provide habitat for a wide variety of animals such as insects and vertebrates [34–36]. Therefore, we suggest that these non-commercial forests play an important role in protecting regional biodiversity. However, biodiversity varies significantly from site to site. For example, tree species richness is clearly lower in pine forest than in mixed and broad-leaved forest, the latter of which have more late successional tree species (Fig 2). This is consistent with other studies that have found higher functional diversity, more late successional species, and high ecosystem functions in broad-leaved forests compared to pine forests [19, 37]. The conservation value of non-commercial forests clearly depends on local environmental conditions and forest type.

### 4.2 Determinants of plant biodiversity

Biodiversity patterns are driven by various factors such as landscape history, environmental variables, and anthropogenic activities [38–40]. Here, in non-commercial forests, we found that plant species richness and composition are mainly driven by forest type and environmental factors, rather than forest age (Figs 2–4; Table 2; Table 4).

Species richness and composition varied significantly among forest types, with more late successional tree species in the broad-leaved forests (Fig 3, Table 3). In eastern China, pine forest, mixed forest, and broad-leaved forest are the main forest types. *Pinus massoniana* is a fast-growing, dominant species during early succession, with a high average importance value (60.9%). The broad-leaved trees in pine forests were largely early successional species, such as *Quercus fabri* and *Liquidambar formosana*. In some nutrient-poor sites (e.g. ridge tops), pine forest will persist for a long time [41]. In some environments (e.g. valleys), shade-tolerant trees (mostly evergreen broad-leaved trees) can increase in abundance and richness while pines die out as succession proceeds. Therefore, evergreen broad-leaved trees such as *Cyclobalanopsis glauca*, *Schima superba*, and *Castanopsis sclerophylla*, dominated broad-leaved forests. Similarly, a study by Wang et al. [42] showed that mixed and broad-leaved forests contain both early and late successional species, together contributing to high species richness. Yuan et al. [43] also found more late successional tree species in mature forests, and a greater representation of fast growing shrub species in pine forests. These patterns pertain not only to taxonomic diversity: functional diversity and ecosystem functions are also reported to be higher in broad-leaved forests than in the other two forest types [19]. Shifting from pine forest to broad-leaved forest is a long-term process, lasting many decades [21], although disturbance such as thinning pine or pine wilt disease should accelerate succession [44]. Hence, our study confirmed the importance of broad-leaved forests for biodiversity conservation in this region.

Environmental factors affect the distribution of plant species and create spatial patterns of biodiversity [45–47]. Our results were similar to some studies in subtropical regions where environmental conditions explained about 20% or less of the variance in species composition [2, 48–50]. In terms of specific environmental variables, we found that elevation and soil depth are especially important in driving species composition patterns (Table 4), highlighting the importance of topography and soil in shaping community structure [51, 52]. The relatively low explanatory power of these models suggest that species composition of non-commercial forests is determined to a considerable degree by other unmeasured factors, such as landscape context, land use history, or climate variables, all of which require further research [39, 53, 54]. Interestingly, species composition among broad-leaved forests was more strongly predicted by environmental factors. This suggests that stochastic colonization and establishment might be more important determinants of species composition in pine and mixed forests [55]. In broad-leaved forests, species might be more strongly filtered by environmental variables [54, 56]. Given the strong compositional differences among forest types, our study also suggests that high habitat heterogeneity supports greater species diversity [57].

Species richness is positively correlated with forest age after disturbance in mixed and broad-leaved forests (Table 2), indicating that species richness can increase rapidly after disturbance. However, species richness in pine forests was not affected, which might due to the long-term persistence of the dominant *Pinus massoniana* tree, which can persist for more than 150 years, potentially preventing colonization by new species [41]. Surprisingly, we didn’t find any effects of forest age on species composition (Fig 4). This result contradicts many previous studies that found directional trends in species composition during succession [58, 59], with more late successional tree species increasing over time [60]. This result might have several causes. First, landscape context can play an important role in driving biodiversity patterns. For example, most of the non-commercial forests in Zhejiang province are fragmented, but we did not evaluate this possible effect [61], which can potentially favor early successional species and therefore counter the expected effect of forest age [62, 63]. Second, the recovery of species richness and composition in secondary forest is generally slow process [64], which might take even more than a hundred years [21]. It is possible that the age differences among our forests (decades) were not sufficient to reveal differences that will emerge over longer periods of time (e.g., centuries). These possibilities are in need of future study.

### 4.3 Implications of future forest management

Anthropogenic activities play an increasingly dominant role in determining forest structure and composition, making forest management an increasingly important global issue [65]. Forest management practices have traditionally paid little attention to biodiversity [66]. Here we focused on biodiversity in non-commercial forests, and our findings provide several implications for forest management.

First, we found that most of this region’s non-commercial forests (i.e., pine dominated) are of relatively low conservation value. Hence, an increase in forest area does not necessarily translate into increasing biodiversity. Biodiversity in newly planted pine forests are much lower than that in old growth broad-leaved forests [7, 8]. Conifer forests dominated by early successional species have expanded considerably in coverage, mainly due to large scale tree plantations establishment in the 1970s [67]. Mature broad-leaved forests are especially important for biodiversity conservation, representing a restoration target, but account for only a small portion of non-commercial forests. Therefore, conservation efforts aimed at biodiversity conservation should focus on promoting attributes found in mature broad-leaved forests [9, 68].

Secondary forests, created by logging in recent decades, are common in eastern China, and to the extent that pine forests can be converted to mixed and broad-leaved forests [22, 43], local biodiversity would be enhanced. However, natural succession is a long-term process, affected by many different factors [21, 69]. Forest management activities can potentially accelerate forest succession. For example, mixed broad-leaved-conifer forest dominated by *Pinus massoniana* in Qiandao Lake National Forest Park of Zhejiang Province, developed rapidly toward succession of evergreen broad-leaved forest after six years of selective cutting of pines [70]. Thinning pine and planting broad-leaved trees is also an effective method of pine forest transformation to broad-leaved forest [10]. Planting can begin with native early successional broad-leaved trees such as *Liquidambar formosana*, which can grow in pine forests (Table 3).

Finally, management activities can be tailored to different forest types (Table 5). For example, while introducing broad-leaved species to pine forests might be necessary, in mixed forests many target species are already present [43], so their growth can simply be enhanced (e.g., by crop tree release and fertilization). For the relatively rare mature broad-leaved forests, strict protection from anthropogenic disturbances can help promote biodiversity conservation.

**Table 5:** Current status and management activities in different forest types in this region.

Forest types	Current status	Possible management activities
Pine forest	Native early successional broad-leaved trees such as *Liquidambar formosana* and *Schima superba* present at low abundance.	Thinning of pine and planting native early successional broad-leaved trees
Mixed forest	Many valuable broad-leaved species such as *Cyclobalanopsis glauca* and *Castanopsis sclerophylla* are present.	Thinning of pine and improving the growth of valuable broad-leaved species (e.g., singling)
Broad-leaved forest	More shade tolerant and late successional tree species such as *Cyclobalanopsis glauca*, *Schima superb*a, and *Castanopsis sclerophylla*	Strict protection from harvesting of mature broad-leaved trees; improvement of the growth of target broad-leaved trees (e.g., singling)

## Supporting information

S1 Table Environmental data of the 600 plots. (XLSX)

S2 Table Species list of the 600 plots. (XLSX)
